# Methotrexate utilization in Rheumatoid arthritis. A register-based cohort-study of treatment re-starts after gabs of at least 90 days

**DOI:** 10.1186/s40064-015-0975-x

**Published:** 2015-05-15

**Authors:** Annette de Thurah, Mette Nørgaard, Kristian Stengaard-Pedersen

**Affiliations:** Department of Rheumatology, Aarhus University Hospital, Nørrebrogade 44, 8000 Aarhus, Denmark; Department of Clinical Epidemiology, Institute of Clinical Medicine, University Hospital, Aarhus, Denmark; Department of Clinical Medicine, Aarhus University, Aarhus, Denmark

**Keywords:** Rheumatoid arthritis, Methotrexate, Drug utilization

## Abstract

**Objective:**

To examine restart of MTX treatment among patients with rheumatoid arthritis (RA) who discontinues treatment, and investigate predictors of restart.

**Methods:**

A cohort study was conducted based on data from medical databases. MTX drug discontinuation was defined as a gap ≥ 90 days from the expiration of one MTX prescription to the redemption of a new one. Kaplan Meier estimates were used to compute the cumulative probability of restarting MTX treatment and Cox proportional hazard to estimate the hazard of return to treatment. A case-crossover analysis compared the frequency of events that could potentially have a transient effect on MTX restart.

**Results:**

Among 788 patients, who started MTX, 299 patients experienced a gap ≥ 90 days. Within 1.4 years 50 % of these patients returned to treatment, and a total of 66 % restarted treatment during follow-up. Concurrent treatment with corticosteroid and disease-modifying antirheumatic drugs (DMARDs) tended to be negatively associated with MTX restart (OR: 0.7(95 % CI: 0.5-1.2) and (OR: 0.7 (95 % CI: 0.4-1.0)). Older patients were less inclined to restart treatment than middle-aged patients (Adjustet HR 0.7 (0.4-1.2)). Patients with a CRP > 300 nmol/L less often restarted MTX than patients with a CRP < 75 nmol/L (adjHR: 0.6 (95 % CI 0.3-1.2)), and men were more inclined to MTX restart than women (adjHR 1.30 (95 % CI 0.9-2.0)).

**Conclusion:**

It is important to support patients in remaining continuous users of MTX. A large proportion of RA patients who discontinued MTX later restarted treatment, but especially patients with high disease activity, old age or co-morbidity were less inclined to restart treatment.

## Background

In rheumatoid arthritis (RA) methotrexate (MTX) is still considered the first drug of choice and early, aggressive and continuous treatment is recommended (Royal College of Physicians 2009; Lopez-Olivo et al. 2014). Sequencing of DMARD has recently been investigated in a systematic review among 503 RA patients in stable disease who were randomized to either continuous treatment or placebo. It was found that patients who remained on effective DMARD had significantly fewer flare episodes than those who discontinued treatment (O’Mahony et al. 2010). Newly, the effectiveness of MTX was once again verified in a meta-analysis (Lopez-Olivo et al. 2014). The same study showed that the MTX discontinuation rate due to adverse effects within 52 weeks were 16 %. In an earlier register based study about MTX compliance we went through a total of 509 medical journals to look for reasons for MTX discontinuation. In this sample approximately 30 % of the patients discontinued MTX at any time during follow-up, and 50 % of this discontinuation were explained by adverse events (AE). The most frequent reported AE was gastrointestinal symptoms (30 %) (de Thurah et al. 2010). It is known, that in chronic diseases such as osteoporosis or chronic gout patients frequently stop and restart treatment for different reasons (Harrold et al. 2010; Brookhart et al. 2007), but it is unclear whether RA patients likewise tend to stop and restart MTX treatment.

In this study we wish to describe the extent to which RA patients, who discontinued MTX treatment for an extensive period, later resumed their treatment and further, to investigate whether clinically important factors such as disease duration, disease activity and side effects could predict return to MTX treatment.

## Methods

### Study population

The study was conducted in the County of Aarhus, Denmark, with a population of approximately 650,000 inhabitants, corresponding to 13 % of the Danish population.

We identified patients with RA through the Danish National Patient Registry (DNPR) using the following ICD-10 codes: M05.3, M05.9, M05.8, M06.0 and M06.9. Our analysis focused on new users of MTX defined as patients with at least one prescription of MTX redeemed between 1 January 1998 and 31 December 2006 and no prescription for MTX redeemed in 1996 and 1997. We retrieved information on MTX use from the regional Pharmaco-Epidemiological Prescription Database (PEPD). All pharmacies in Denmark are equipped with electronic accounting systems that are used to secure reimbursement from the National Health Service, which funds a variable proportion of the cost of prescribed medicine for all Danish citizens. Data are transferred to the PEPD, which thus covers all reimbursed drugs at the level of the individual user. The database includes: *1)* information on type of drug according to the Anatomical Therapeutical Chemical Classification System (ATC), *2)* the date when the prescription was filled, *3)* the patients civil registry number (CPR number), *4)* packing size and the number of pills in each package (always 100 tablets of 2.5-mg), and *5)* the amount of drug according to number of defined daily doses (DDD) (WHO 2015). The ATC codes for MTX are: L01BA01 and L04AX03 (WHO 2015). In Denmark the physicians and pharmacists are regulated separately which means a separation of prescribing and dispensing of drugs. Hence, the pharmacists must give the exact treatment prescribed by the doctor. Only oral MTX is included in the PEDP.

### Definition of treatment gaps and return to treatment

No standard definition exist on the length of a gap that qualifies as drug discontinuation based on pharmacy records. Our definition is based on the literature saying that the variation in persistence is most pronounced for treatment gaps between 9–90 days, whereas treatment gaps between 90–360 days only have minor influence on the percentage of persistent patients (Van Wijk et al. 2006). Thus, in conjunction with others (Grijalva et al. 2007), we defined MTX drug discontinuation as a gap ≥ 90 days from the expiration of one MTX prescription to the redemption of a new one. We accordingly defined return to treatment as redemption of an MTX prescription after a gap ≥ 90 days.

As the PEPD does not contain data on the exact doses, but only the defined daily dose (DDD), we reviewed the medical records of all patients and retrieved the prescribed daily dose (PDD) defined as the dose the patients were receiving 6 months after stat of treatment (Steiner & Prochazka 1997). The PDD was used in the calculation of the gabs, and the 90-days gab in treatment could occur at any time during follow-up.

### Other covariates

We defined disease duration as the time from the date of first RA diagnosis in the NPR until the date of the first MTX prescription in the PEPD.

From the County Clinical Biochemistry Registry we retrieved the latest C-reactive protein (CRP), alanine aminotransferase (ALAT) and hemoglobin (Hb) analysis performed prior to a gap ≥ 90 days. We regarded CRP as surrogate marker for disease severity (Yildirim et al. 2004). We defined liver enzyme evaluation as ALAT two times the upper limit of normal (Saag et al. 2008). Low hemoglobin was defined as: female: < 7.4 mmol/l, male: < 8.4 mmol/l.

Co-morbidity was assessed by Charlsons Co-morbidity Index (CCI) (Charlson et al. 1987) and patients were categorized as having co-morbidity if, since 1997, they had at least one of the 19 CCI diagnoses recorded in the NRP. Through the PEPD we retrieved information on concurrent use of corticosteroids (H02AB06) and other conventional DMARDs: sulfasalazine (A07EC01) and cloroquine (P01BA0, P01BA01).

In Denmark, biological DMARDs are managed through hospital and thus are not a part of the PEDP. We collected data on Etanercept (ATC L04AA11), Infliximab (ATC L04AA12) and Adalimumab (ATC L04AA17) through the Danish Danbio register (Hetland et al. 2005). However, as most patients switched to subcutanous MTX treatment before initiating anti-TNF-alfa treatment to few cases were left for analysis. Leflunmide, goldsalt, penicillamin and cyclosporine are very seldom used in RA in Denmark.

### Statistics

Included patients were followed from the date they experienced a first gap, until a new prescription was filled or the end of the study period, whichever came first. Kaplan Meier estimates were used to estimate the cumulative probability of MTX restart, and Cox proportional hazard to estimate the hazard ratio of restart. Case-crossover analysis was used to compare the frequency of factors with potential effect on MTX restart (CRP, hemoglobin, ALAT, concurrent use of conventional DMARDs and corticosteroids). The frequency was estimated within 60 days before MTX restart (the hazard period) and compared with the frequency 61–180 days before MTX restart (the control period) (Schneeweiss et al. 1997).

## Results

**Fig. 1:**
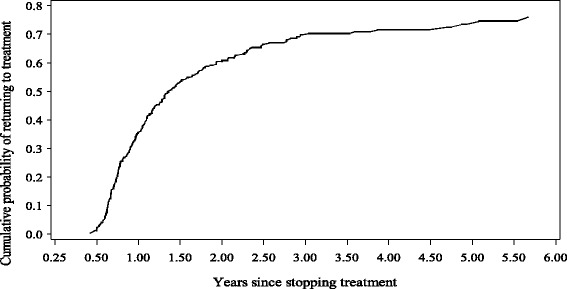
1-Kaplan Meier estimate of the cumulative probability of returning to Methotrexate treatment after a gap ≥ 90 days among 299 rheumatoid arthritis patients in the County of Aarhus, Denmark 1996–2006

During the study period 788 patients started MTX treatment and 299 patients (38 %) experienced a gap ≥ 90 days. These patients were followed for a median of 352 days (range 168–2067) from 90 days after their last MTX prescription had expired (Fig. 1).

The percentage of younger patients (<55 years) who experienced a treatment gap was 36 % vs. 28 % among those who did not. Patients with and without a gab had similar distribution of sex, co-morbidity, disease duration, concurrent medication, and disease activity.

Within 1.4 years 50 % of the patients restarted MTX, and within 3 years 66 % of the patients had restarted treatment (Fig. 1). None of the factors in the case-cross over analysis were associated with MTX restart. Only concurrent treatment with either corticosteroid or DMARDs, showed a tendency towards a negative association to MTX restart (OR 0.7 (95%CI 0.4;1.1) and OR 0.7 (95 % CI.0.4;1.2) respectively) (Table 1).

**Table 1:** Crude and adjusted hazard ratios (HR) with 95 % confidence interval (CI) for methotrexate restart after a treatment gab ≥ 90 days among 299 patients with rheumatoid arthritis in the County of Aarhus, Denmark 1998-2006

Gender	Male	203 (67.9)	1.3 (0.9;1.9)	1.3 (0.9;2.0)
	Female	96 (32.1)	1	1
Age	<55 years	109 (36.5)	0.8 (0.5;1.3)	0.9 (0.6;1.7)
	55-67 years	92 (30.7)	1	1
	>67 years	98 (32.8)	0.7 (0.4;1.1)	0.7 (0.4;1.2)
C-reactive protein	< 75 nmol/L	154 (51.5)	1	1
	76-300 nmol/L	78 (26.1)	1.0 (0.6;1.5)	1.1(0.7;1.8)
	>300 nmol/L	60 (20.1)	0.5 (0.3;0.9)	0.6 (0.3;1.2)
	Missing	7 (2.3)	-	-
Hemoglobin	Low	89 (29.8)	1.4 (0.9;2.2)	1.0 (0.6;1.8)
	Normal	203 (67.9)	1	1
	Missing	7 (2.3)	-	-
Co-morbidity	0	209 (69.9)	1	1
	> = 1	90 (30.1)	0.8 (0.5;1.2)	0.8 (0.5;1.3)
Concurrent DMARD	Yes	110 (36.8)	0.7 (0.5;1.0)	0.8 (0.5; 1.2)
	No	189 (63.2)	1	1
Corticosteroids	Yes	100 (33.4)	0.5 (0.3,0.7)	0.7 (0.4;1.1)
	No	189 (66.6)	1	1

Data suggested that older patients less often restarted MTX treatment compared with middle-aged patients (adjusted hazard ratio (adjHR): 0.7 (95 % confidence interval (CI) 0.4-1.2)). A similar pattern was seen for patients with co-morbidities compared with patients without co-morbidity (adjHR: 0.8 (95 % CI 0.5-1.2)). Patients with a CRP > 300 nmol/L less often restarted MTX than patients with a CRP < 75 nmol/L (adjHR: 0.6 (95 % CI 0.3-1.2)), and men were more inclined to MTX restart than women (adjHR 1.30 (95 % CI 0.9-2.0)). However, none of the predictors reached statistical significance (Table 1).

## Discussion

The present study found that among RA patients who discontinued MTX treatment ≥ 90 days, 66 % restarted treatment within 3 years of follow-up, suggesting MTX utilization among RA patients to resemble medication-taking patterns in line with that seen among patients with other chronic diseases (Harrold et al. 2010; Brookhart et al. 2007).

In daily clinical practise, an inadequate response to MTX may be followed by switching to an alternative DMARD (Royal College of Physicians 2009), and in line with this we found the use of either corticosteroids or concurrent DMARDs to deter MTX restart. Similar to others, (Schmajuk et al. 2010; Solomon et al. 2014) we also found that patients with increasing age and high levels of co-morbidity were less prone to restart MTX treatment. In a previous study about MTX compliance among patients with RA we found men to be more compliant compared to women (de Thurah et al. 2010). In keeping with this finding the current study also found men to be more inclined to MTX restart than women.

The present study has limitations that merit further discussion.

First of all, it is a challenge to determine the length of the gap that defines drug discontinuation, and no standard definition exists (Steiner & Prochazka 1997). However, a Dutch cohort study investigating the variation in persistence with drugs found that the variation in persistence was most pronounced for treatment gaps between 9–90 days, whereas treatment gaps between 90–360 days only had minor influence on the percentage of persistent patients (Van Wijk et al. 2006). Hence, we choose ≥ 90 days as cut off in the definition of a treatment gab.

Secondly, we lacked data on level of pain and functional disability that could potentially have influenced MTX utilization more directly and further, we were unable to determine whether gaps in MTX treatment were doctor prescribed, i.e. due to side effects (Nikiphorou et al. 2014). In an earlier register based study about MTX compliance we went through a total of 509 medical journals to look for reasons to MTX discontinuation (de Thurah et al. 2010). In this sample approximately 30 % of the patients discontinued MTX at any time during follow-up, and 50 % of this discontinuation were explained by AE’s. The most frequent reported AE was gastrointestinal symptoms (30 %). In general, the documentation of data in this area tend to be insufficient in the medical records, potentially leading to differential misclassification, as reasons for doctor prescribed or, especially, patient initiated breaks are only sporadic documented. In a recent meta-analysis MTX discontinuation rate due to AE’s within 52 weeks were found to be 16 % (Lopez-Olivo et al. 2014), and our finding, that toxicity rather than lack of effect were the main reason for MTX discontinuation is in agreement with this observation (Felson et al. 1992).

In conclusion, our findings suggest that RA patients frequently stop and restart treatment. We found that especially patients with high disease activity and old age or co-morbidity were less inclined to restart treatment. Hence, addressing MTX utilization and side effects routinely is important in daily clinical practise in order to help patients to remain continuous users.
